# Strong population structure but no equilibrium yet: Genetic connectivity and phylogeography in the kelp *Saccharina latissima* (Laminariales, Phaeophyta)

**DOI:** 10.1002/ece3.3968

**Published:** 2018-04-02

**Authors:** Pieternella C. Luttikhuizen, Freek H. M. van den Heuvel, Céline Rebours, Harry J. Witte, Judith D. L. van Bleijswijk, Klaas Timmermans

**Affiliations:** ^1^ Department of Coastal Systems NIOZ Royal Netherlands Institute for Sea Research and Utrecht University Den Burg The Netherlands; ^2^ Hortimare B.V. Heerhugowaard The Netherlands; ^3^ Norwegian Institute of Bioeconomy Research (NIBIO) Ås Norway; ^4^ Møreforsking Ålesund AS Ålesund Norway; ^5^ Department of Marine Microbiology NIOZ Royal Netherlands Institute for Sea Research and Utrecht University Den Burg The Netherlands; ^6^ Department of Estuarine and Delta Systems NIOZ Royal Netherlands Institute for Sea Research and Utrecht University Yerseke The Netherlands

**Keywords:** aquaculture, connectivity, conservation genetics, phylogeography, population genetics—empirical

## Abstract

Kelp aquaculture is globally developing steadily as human food source, along with other applications. One of the newer crop species is *Saccharina latissima*, a northern hemisphere kelp inhabiting temperate to arctic rocky shores. To protect and document its natural genetic variation at the onset of this novel aquaculture, as well as increase knowledge on its taxonomy and phylogeography, we collected new genetic data, both nuclear and mitochondrial, and combined it with previous knowledge to estimate genetic connectivity and infer colonization history. Isolation‐with‐migration coalescent analyses demonstrate that gene flow among the sampled locations is virtually nonexistent. An updated scenario for the origin and colonization history of *S. latissima* is developed as follows: We propose that the species (or species complex) originated in the northwest Pacific, crossed to the northeast Pacific in the Miocene, and then crossed the Bering Strait after its opening ~5.5 Ma into the Arctic and northeast Atlantic. It subsequently crossed the Atlantic from east to west. During the Pleistocene, it was compressed in the south with evidence for northern refugia in Europe. Postglacial recolonization led to secondary contact in the Canadian Arctic. *Saccharina cichorioides* is shown to probably belong to the *S. latissima* species complex and to derive from ancestral populations in the Asian North Pacific. Our novel approach of comparing inferred gene flow based on coalescent analysis versus Wright's island model suggests that equilibrium levels of differentiation have not yet been reached in Europe and, hence, that genetic differentiation is expected to increase further if populations are left undisturbed.

## 1 INTRODUCTION

**Figure 1:**
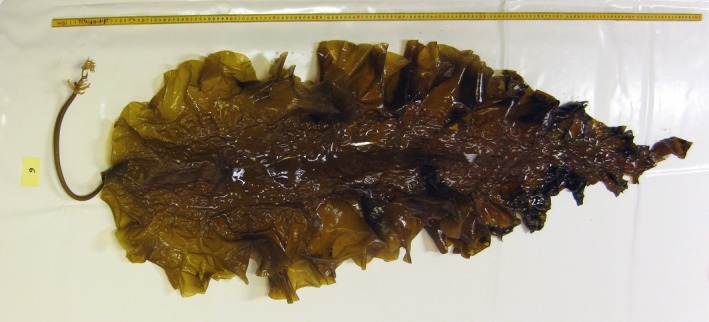
Sea belt *Saccharina latissima* with centimeter scale. Photograph credit: Céline Rebours

Aquaculture is a growing portion of the world's seafood production (FAO 2016). Global production stemming from aquaculture was 40% in 2005 (Asche, 2008) and surpassed capture fisheries in 2013 (FAO 2016). Aquatic plants follow this trend: Aquatic plant aquaculture production has increased from 13.5 million tonnes in 2005 to 27.3 million tonnes in 2014 (FAO 2016). Kelp and components of kelp have a broad range of applications, including food for humans, soil fertilizer, animal feed, cosmetics and, potentially, biofuel (Fernand et al., 2017). While kelp has been taken from wild stands for centuries in, for example, Japan and China, cultivating them has allowed for increased production. At the same time, the demand for kelp has spread around the globe (McHugh & FAO, 2003). Risks associated with kelp farming include the introduction of alien species and the loss of natural genetic variation. An example of alien species introduction is the Asian kelp *Undaria pinnatifida*. This species has been introduced for farming throughout the world, has escaped to the wild in numerous places, and interferes with local species (Dellatorre, Amoroso, Saravia, & Orensanz, 2014; Peteiro, Sanchez, & Martinez, 2016; Veiga, Torres, Rubal, Troncoso, & Sousa‐Pinto, 2014). An example of the loss of genetic variation is *Saccharina japonica*. Native to Asia, this kelp species has a long history of traditional wild stand utilization. In the present day, it has a shallow population genetic structure in its native range, which has been attributed in part to anthropogenic interference (Zhang et al., 2015). It is important to protect natural interspecific and intraspecific diversity in kelp both for resilient ecosystem functioning and for providing genetic material for use in domestication (Loureiro, Gachon, & Rebours, 2015). To protect natural variation, the first task is to document it. Therefore, we here present data on phylogeography and genetic connectivity for the cold water kelp species *Saccharina latissima* (Linnaeus) C.E. Lane, C. Mayes, L.D. Druehl & G.W. Saunders (“sea belt,” Figure 1) whose cultivation is developing in the northern Atlantic Ocean (Azevedo, Marinho, Silva, & Sousa‐Pinto, 2016; Forbord et al., 2012; Reid et al., 2013).

The distribution range of *S. latissima*, which inhabits the lower intertidal of rocky shores down to a depth of approximately 30 m, encompasses both sides of the North Atlantic, the northeast Pacific as well as the Arctic Ocean (Bolton, 2010). The family it belongs to, the Laminariaceae, originated in the temperate North Pacific in the Miocene, subsequently splitting into several genera, at least four of which colonized the Arctic and North Atlantic following the opening of the Bering Strait about 5.5 Mya (Bolton, 2010; Marincovich & Gladenkov, 1999). The Pleistocene ice ages shifted the distribution of kelp southward and isolated the Pacific from the Atlantic basin, while at the same time, there is evidence to suggest that kelp forests were far more extensive and productive during glacial maxima than in the present day (Erlandson et al., 2007). With regard to *S. latissima*, it has been hypothesized that the Last Glacial Maximum isolated populations in the northeast Pacific, northwest Atlantic and northeast Atlantic, with subsequent recolonization from Europe and secondary contact in the Arctic (McDevit & Saunders, 2010). Given that scenario, present‐day European *S. latissima* populations would originate from a compressed southern European source population, which should be reflected in the distribution of the taxon's molecular variation.

Brown algal kelp species have sufficiently strong dispersal barriers to allow inference of their biogeographies from present‐day distributions (Bolton, 2010). This trend of relatively low dispersal capacity continues at smaller—intraspecific—spatial scales. Significant and strong population structure is typically present among kelp populations (Billot, Engel, Rousvoal, Kloareg, & Valero, 2003; Johansson et al., 2015; Uwai et al., 2006). However, low levels of population structure have been documented for regions with strong boundary currents (Coleman et al., 2011), populations resulting from human‐mediated invasion events (Grulois, Leveque, & Viard, 2011), and natural ranges with high levels of cultivation and/or transport activities (Shan et al., 2012; Zhang et al., 2015). Therefore, it is important to document the undisturbed genomic variation in a kelp taxon prior to its exploitation. In‐depth knowledge of the starting situation will help to evaluate transport and cultivation activities. Significant population structure has recently been reported for *S. latissima* in the area connecting the Baltic Sea with the North Sea (Nielsen et al., 2016), as well as at a larger scale across Europe (Guzinski, Mauger, Cock, & Valero, 2016). However, genetic connectivity in *S. latissima* has not yet been estimated.

The mechanisms limiting effective dispersal by natural means in kelp are most likely related to life cycle characteristics rather than to specifics of the environment. Dispersal mechanisms in kelp include floating of dislodged sporophytes or sporophyte fragments (drifters) and passive dispersal by meiospores. Pneumatocysts are gas‐filled bladders that assist in keeping sporophyte blades near the sea surface for photosynthesis, while other species float well without pneumatocysts. Drifting may result in the capacity for long‐distance dispersal (Thomas, 2002), but studies have shown that most drifters do not travel far (López, Macaya, Tala, Tellier, & Thiel, 2017; Reed, Schroeter, & Raimondi, 2004). Meiospore dispersal distance varies with species and is rarely more than 1 km (Fredriksen, Sjotun, Lein, & Rueness, 1995; Gaylord, Reed, Raimondi, & Washburn, 2006). An important factor in effective meiospore dispersal is that following settlement, high densities are required for successful fertilization (Reed, 1990a,1990b).

Spatial genetic data can be used to make inferences about dispersal, although many assumptions underlie the various approaches that have been developed (Broquet & Petit, 2009; Samarasin, Shuter, Wright, & Rodd, 2017). The classical Wright's island model (Wright, 1931) evaluates the genetic differentiation measure *F*_ST_ in terms of dispersal in the hypothetical case of “islands” of equal population sizes and equal migration rates for all possible island pairs. However, it is not the unrealistic landscape model which is the main problem for inferring genetic connectivity in many species, but rather the assumption that equilibrium F_ST_ has been reached. The latter assumption is particularly unrealistic for many marine taxa which are often characterized by (extremely) large populations (e.g., kelp forests) (Hellberg, 2009). The time it takes after initial population subdivision to reach an equilibrium level of genetic differentiation depends primarily on effective population size (Crow & Aoki, 1984). Assuming equilibrium in such cases will lead to overestimation of inferred dispersal rates. We therefore use a coalescent approach for estimating genetic connectivity in *S. latissima* which estimates population divergence time in conjunction with migration rates and mutation, and hence does not depend on assuming equilibrium between isolation and migration (Hey, 2010; Hey & Nielsen, 2004).

The aim of this study was to reconstruct biogeographic history of *S. latissima* and to estimate genetic connectivity within the European range of its distribution. To do this, we sequenced a portion of the mitochondrial cytochrome‐*c*‐oxidase I gene (for compatibility with earlier studies) and genotyped novel EST (expressed sequence tag)‐derived microsatellites. For both phylogeographic inference and genetic connectivity estimation, we took a coalescent approach to simultaneously estimate migration and timing of differentiation, and to allow for the possibility that equilibrium between isolation and migration may not yet have been reached (Hey & Nielsen, 2004; Nielsen & Wakeley, 2001).

## 2 METHODS

### 2.1 Field sampling and DNA purification

**Figure 2:**
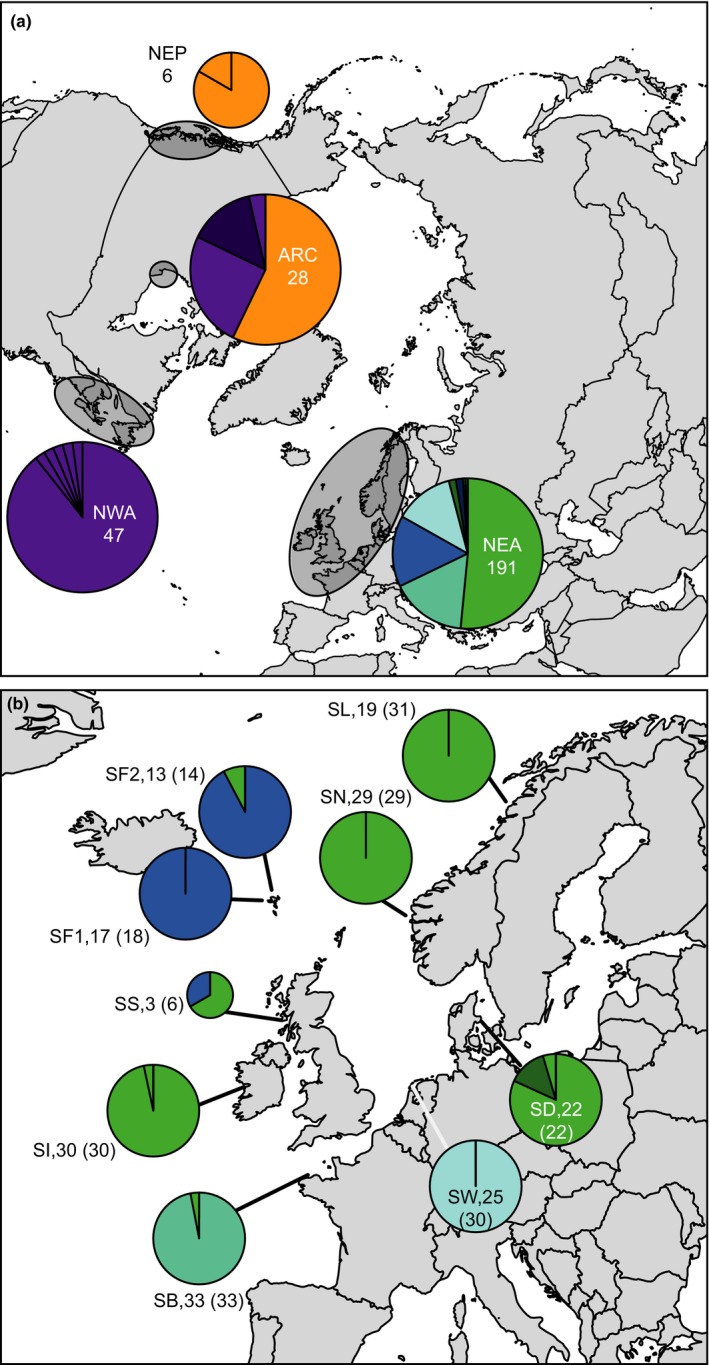
Maps of study area showing geographical distribution of COI haplotypes in *Saccharina latissima*: (a) northern hemisphere view; (b) close‐up of northeast Atlantic. In top panel, sampling locations are indicated by transparent ovals inside each pie chart location abbreviation (see Table 1) and sample sizes are shown. In bottom panel, sampling locations are shown by lines; next to pie charts location abbreviations, COI sample size and microsatellite sample size (in brackets) are shown. Haplotype relationships can be seen in Figure 3

Samples for *Saccharina latissima* (213 individuals) were obtained from eight locations along northwestern European coasts (Table 1, Figure 2b). The material was collected from wild populations, with the exception of 14 individuals from the Faroe Islands, which originate from culture lines. Sporophyte material was dried and DNA was extracted using a modified hexadecyltrimethylammonium bromide (CTAB) extraction protocol in combination with the Mammalian Genomic DNA Purification Kit (Sigma‐Aldrich, USA). After purification, DNA extracts were cleaned with the One Step PCR Inhibitor Removal Kit (Zymo Research, USA).

**Table 1:** Summary of sampling locations and genetic diversity in *Saccharina latissima* samples

	Location	Sampling date	Coordinates	COI				Microsatellites	
				*N*	*N*_h_	π	*h*	*N*	*F*_IS_
SL	Bodø, Norway	2 October 2013	67°04′27″N 14°06′43″E	19	1	0 ± 0	0 ± 0	31	−0.0826
SN	Solund, Norway	September/October 2013	61°12′50″N 4°54′02″E	29	1	0 ± 0	0 ± 0	29	0.131
SF1	Faroe Islands, wild	9 February 2015	62°15′21″N 6°57′14″W	17	1	0 ± 0	0 ± 0	18	−0.0986
SF2	Faroe Islands, culture	idem	idem	13	2	0.000237 ± 0.000396	0.1538 ± 0.1261	14	0.0634
SS	Oban, Scotland, UK	4 March 2014	56°23′52″N 5°30′59″W	3	2	0.001027 ± 0.001281	0.6667 ± 0.3143	6	n.a.
SD	Grenaa, Denmark	21 October 2014	56°23′52″N 10°55′14″E	22	3	0.000520 ± 0.000599	0.3247 ± 0.1173	22	0.173
SW	Marsdiep, the Netherlands	February/August 2013	53°00′07″N 4°47′14″E	25	1	0 ± 0	0 ± 0	30	0.383
SI	Galway, Ireland	15 July 2014	53°14′24″N 9°18′35″W	30	2	0.000103 ± 0.000243	0.0667 ± 0.0613	30	−0.0124
SB	Brittany, France	8 May 2014	48°42′30″N 3°49′23″W	33	2	0.000093 ± 0.000230	0.0606 ± 0.0564	33	0.116
				191	7			213	

*N*, number of individuals genotyped; *N*_h_, number of haplotypes; π, nucleotide diversity; *h*, haplotype diversity; *F*_IS_, inbreeding coefficient.

### 2.2 DNA sequencing

Custom primers were designed to amplify a portion of the mitochondrial gene cytochrome‐*c*‐oxidase I (COI) (kelpCOIf: CATCGGTTATTAGCTCKTCA; kelpCOIr: 5′‐ TGATACAAWACAGGATCACCAC‐3′) based on an alignment of COI for kelp species sourced from Genbank. The PCR mixture consisting of 1 μl of DNA template (1:10 diluted), 0.2 mmol/L dNTP, 1× Taq buffer, 0.5 μmol/L of each primer, 1 μl Bovine Serum Albumine (BSA, 20 mg/ml), and 1.25 unit Taq polymerase (BiothermPlus, GeneCraft, Germany) in a total volume of 50 μl was run on a BioRad T100 thermocycler (BioRad, USA) with initial denaturation at 94°C for 4 min, 35 cycles at 94°C for 30 s, 40°C annealing for 30 s, extension at 72°C for 40 s, and final extension at 72°C for 10 min. A 623 basepair portion of COI was successfully amplified for a total of 191 individual kelp samples and sequenced at Baseclear B.V. (Leiden, the Netherlands). Sequence data were edited and aligned using BioEdit v7.2.5 (Hall, 1999).

### 2.3 Microsatellite fragment analysis

Microsatellite DNA markers were developed based on transcriptome no. SRR305166 (Genbank, Heinrich, Valentin, Frickenhaus, John, and Wiencke, 2012) by searching for dinucleotide and trinucleotide repeats. Primers were designed for 42 potential microsatellite markers and tested on eight different DNAs for consistent amplification (Table S1). Those primer pairs that amplified consistently were tested for repeatable genotyping and polymorphism, retaining a final ten markers (Table S1). Each forward primer was extended at the 5′ end with an adapter (5′‐CACGACGTTGTAAAACGAC‐3′) for annealing during amplification to a fluorescent label with the same extension. Microsatellite PCR's consisting of 1 μl of DNA template (1:10 diluted), 0.2 mmol/L dNTP, 1× Taq buffer, 0.5 μmol/L of each primer, 1 μmol/L fluorescent label, 0.4 μl BSA (20 mg/ml), and 0.5 unit Taq polymerase (BiothermPlus, GeneCraft, Germany) in a total volume of 20 μl were run on a BioRad T100 thermocycler (BioRad, USA) with initial denaturation at 94°C for 4 min, 41 cycles at 94°C for 30 s, 52°C annealing for 30 s, extension at 72°C for 40 s and final extension at 72°C for 10 min. Electrophoresis was carried out on ABI capillary sequencers at Baseclear B.V. (Leiden, the Netherlands; Table S2) and the raw data processed using GeneMapper (Applied Biosystems, v4.0).

### 2.4 Data analysis

Analyses of molecular variance (AMOVA) were carried out in Arlequin 3.5.1.2 (Excoffier & Lischer, 2010). Significance levels were Bonferroni adjusted to account for multiple testing in pairwise comparisons. Microsatellite DNA variation among kelp individuals was visualized by principal coordinates analysis in GenAlEx 6.5 (Peakall & Smouse, 2012). Genetic distances were estimated with MEGA 6 (Tamura et al., 2011) and a minimum spanning network among haplotypes was constructed using the R package Pegas version 0.8‐2 (Paradis, 2010).

#### 2.4.1 Genetic connectivity analyses

Historical population subdivision times were estimated by running coalescent simulations in IMa2 (Hey, 2010). Only pairs of population samples were examined, because we were interested in the order of colonization events and a population tree could therefore not be a priori defined. Coalescent simulations consisted of three independent runs for each comparison, each run consisting of ten Markov Chain Monte Carlo chains with geometric heating (h1 = 0.99, h2 = 0.75) of two million steps after an initial burn‐in period of five million steps. Convergence of parameter distributions was ensured by: examining effective sample size (ESS) values, autocorrelation values and chain swapping, checking trend line plots for the absence of trends, and by comparing parameter estimates generated from the genealogies produced during the first and second half of runs.

Coalescent analyses were carried out for two spatial scales. Three pairs of samples were analyzed for the larger spatial scale of the North Atlantic Ocean (mitochondrial sequences only): among the Faroe Islands (SF, this study), Brittany (SB, this study), and northwest Atlantic (NWA, combined data from Atlantic Canada, and Maine and Rhode Island in the USA from McDevit and Saunders (2010)). The northeast Pacific (NEP) from McDevit and Saunders (2010) could not be included because of its small sample size (*N* = 6), nor the Arctic data from Churchill, Manitoba, because that area is a secondary contact zone for *S. latissima* between NEP and NWA (McDevit & Saunders, 2010), which does not fit the isolation‐with‐migration model. A further 21 pairwise comparisons were carried out at the smaller, intra‐European spatial scale based on the microsatellite data: among the samples SL, SN, SF1, SD, SW, SI, and SB (see Table 1). To simplify the model for the larger spatial scale, migration rate was set to zero, which is a reasonable assumption based on the strong level of differentiation present among the three samples analyzed at this scale (e.g., no shared haplotypes). For the intra‐European scale, full isolation‐with‐migration analyses were conducted, which was more feasible for those comparisons because of the multilocus data.

Molecular clocks in the brown algae are difficult to calibrate directly, because a good fossil record for these soft‐bodied organisms does not exist. First, we follow the rationale by Hoarau, Coyer, Veldsink, Stam, and Olsen (2007), that diatoms are the closest relatives to the phaeophyta with a good fossil record, and have comparable generation times. Hence, molecular clocks are likely to be similar. Comparing COI in *Haslea ostrearia* (Genbank accession number HE995416) and *Skeletonema ardeus* (KM202114), which share a common ancestor less than 65–87 million years ago (Medlin, Kooistra, Gersonde, & Wellbrock, 1996; Sorhannus & Fox, 1999), we arrive at a p‐distance of 0.199. This amounts to a divergence rate of 0.229%–0.306% per million years, and hence a minimum substitution rate of 7.13 × 10^−7^ per year per 623 bp locus. Second, we follow the approach in Zhang et al. (2015) which is based on the assumption that *Saccharina japonica* and *S. augustata* speciated 4.84 million years ago. Comparing representative COI sequences for these two taxa (AP011493 and AP011498), we find a p‐distance of 0.04633 and a divergence rate of 0.9572% per million years. This amounts to a maximum substitution rate of 2.98 × 10^−6^ per year per 623 bp locus. We used the average between this minimum and maximum molecular clock rate as well as the range in the IMa2 simulations. For the microsatellites, we used a mutation rate of 0.001564, which is the average across all multicellular taxa for which multiple loci were studied as reported in Brohede (2003). This is a simplification as mutation rate in microsatellites is known to depend on many factors, including repeat length and number, heterozygosity, and DNA repair mechanisms (Bhargava & Fuentes, 2010); no microsatellite mutation rates are known to date for brown algae and therefore results will need to be evaluated with caution.

#### 2.4.2 Comparing equilibrium and nonequilibrium inferences

Estimates of genetic connectivity were also made based on Wright's island model (Wright, 1931) which depends on, among others, the assumption that equilibrium between migration, drift, and mutation has been reached. Those equilibrium‐based estimates were compared with those based on coalescent analyses as described above, which do not assume equilibrium has been reached, by correlation, taking the average between the two‐two‐way migration rates for the nonequilibrium estimates.

## 3 RESULTS

### 3.1 Genetic variability

**Figure 3:**
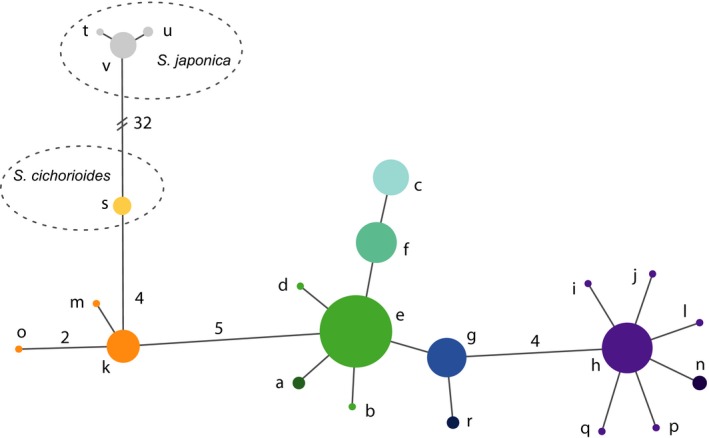
Minimum spanning network among COI haplotypes (623 bp) for *Saccharina latissima*. Haplotypes a–g from this study; h–r from (McDevit & Saunders, 2010), s–v from (Balakirev et al., 2012)

Seven different haplotypes with six variable sites were detected among the COI sequences (Genbank accession numbers: MF447855‐MF447861). These haplotypes (a–g) were closely related, as can be seen in the minimum spanning network (Figure 3). Despite this low level of variability, the sampling locations differed markedly in their haplotype frequencies (Figure 2, Table S3). These newly collected data were combined with published sequence data on the same locus for the same species from McDevit and Saunders (2010) and from the closely related species *Saccharina cichorioides* and *S. japonica* from Balakirev, Krupnova, and Ayala (2012). After cropping to the same length, this yielded an additional 111 sequences which collapsed to 15 haplotypes (h–v, Figure 3, Table S3). Variability of microsatellite markers varied among markers from two to seven alleles with one highly variable locus (Lat19) with 27 alleles (Table S1).

### 3.2 Population structure

**Figure 4:**
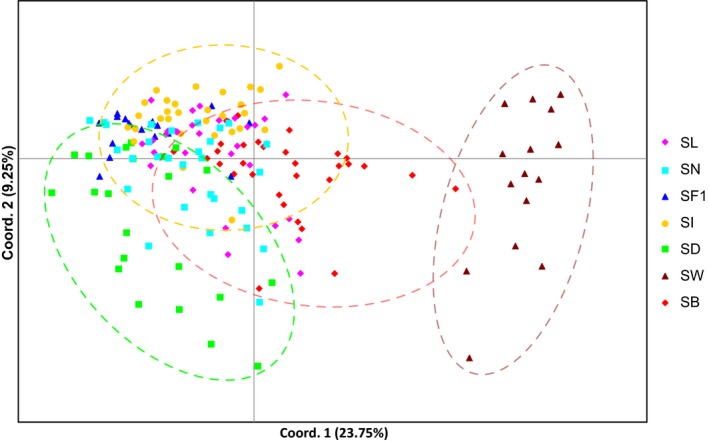
Principal coordinates analysis on microsatellite data for *Saccharina latissima*. For abbreviations of sampling locations, see Table 1

Samples from Scotland were omitted from all population analyses because of small sample size. With regard to the Faroe Islands samples, analyses were performed both with and without individuals sampled from culture lines to assess any differences. Strong and significant genetic differentiation between samples was found in a majority of cases. The COI sequences showed a lack of differentiation among the samples from Denmark, Ireland, southern and northern Norway, while all other comparisons were strongly and significantly differentiated (Table 2). Genetic differentiation based on microsatellite markers was significant for all comparisons, regardless of whether samples from Faroe culture lines were taken into account (Table 2). The overall level of population differentiation was strong and significant (*F*_ST_ = 0.268 (*p *<* *.001)). This can also be seen visually from principal coordinates analysis of the microsatellite data (Figure 4).

**Table 2:** Analysis of molecular variance (AMOVA): fixation indices (top panel) and pairwise sample comparisons (bottom panel: below diagonal for microsatellite markers, above diagonal for COI sequences). In brackets are values for AMOVA excluding Faroe culture line (CL) samples. All fixation indices are significantly larger than zero (top panel, in bold). Fixation indices for pairwise population comparisons in bold (bottom panel) are significantly different from zero after Bonferroni correction. MV, percentage of molecular variance

Overall differentiation		
		MV
Φ_ST_	**0.936 (0.932)**	94% (93%)
F_ST_	**0.318 (0.267)**	32% (27%)
F_IS_	**0.090 (0.275)**	62% (53%)
F_IT_	**0.379 (0.468)**	6% (20%)

Pairwise comparisons							
	SB	SD	SF	SI	SL	SN	SW
SB	—	**0.849**	**0.967 (0.980)**	**0.937**	**0.960**	**0.967**	**0.967**
SD	**0.177**	—	**0.840 (0.844)**	0.075	0.061	0.093	**0.927**
SF	**0.153 (0.176)**	**0.071 (0.052)**	—	**0.933 (0.960)**	**0.958 (1.000)**	**0.965 (1.000)**	**0.988 (1.000)**
SI	**0.106**	**0.140**	**0.135 (0.121)**	—	−0.016	−0.00115	**0.982**
SL	**0.103**	**0.097**	**0.056 (0.092)**	**0.064**	—	0.000	**1.000**
SN	**0.096**	**0.084**	**0.098 (0.093)**	**0.087**	**0.050**	—	**1.000**
SW	**0.360**	**0.564**	**0.454 (0.549)**	**0.454**	**0.410**	**0.469**	—

### 3.3 Colonization history

Results from coalescent analyses at the larger spatial scale of the North Atlantic basin based on mitochondrial COI sequences are shown in Table 3A and Figure S1. Results among independent runs with the same data and settings were highly reproducible (Figure S1). Present‐day effective population sizes were estimated to range between 114 and 788 per population, while for ancestral effective population sizes posterior probability densities distributions were too flat to allow meaningful inferences (Figure S1). Population subdivision time for the comparisons between eastern and western North Atlantic was estimated to be between 1.22 and 1.68 million years ago (Mya). Because the eastern North Atlantic haplotypes are more closely related to the western North Pacific haplotypes than the western North Atlantic haplotypes are (Figure 3), it is more likely that the European side of the North Atlantic was colonized first by *S. latissima* and that the Northwest Atlantic side was subsequently colonized across the Atlantic from Europe to North America. Moreover, haplotype g, which was found only at outer Atlantic locations Scotland and Faroe Islands, is situated between the European and the North American haplotypes in the minimum spanning network, suggesting that the present‐day patterns are partially a remnant of this colonization process.

**Table 3:** Maximum posterior probability estimates in demographic units for isolation‐with‐migration simulations: (A) large‐scale comparisons across the Atlantic Ocean (mitochondrial COI sequences), (B) small‐scale comparisons among European samples (microsatellite markers). For details of simulations, see text. *N*_*x*_ = thousands of individuals in population represented by sample *x* (estimated effective population size); *T* = population subdivision time (years); *m*_a>b_ = forward‐in‐time migration rate from a (left sample in first column) to b (right sample in first column) (numbers of individuals per generation); n.e. = not estimated

A					
Sample pair	*T*	*N*_a_	*N*_b_	*m*_a>b_	*m*_b>a_
SB vs. SF	0.174 × 10^6^ (0.171–0.176)	126 (124–129)	121 (116–124)	n.e.	n.e.
SB vs. NWA	1.29 × 10^6^ (1.22–1.32)	145 (136–154)	788 (776–797)	n.e.	n.e.
SF vs. NWA	1.61 × 10^6^ (1.55–1.68)	114 (109–120)	780 (778–783)	n.e.	n.e.
B					
SL vs. SN	7	39.37	18.12	3.07	0.144
SL vs. SF1	77	13.12	50.62	0.430	0.0294
SL vs. SD	4	4.38	25.62	0.0911	2.25
SL vs. SW	122	59.37	0.63	0.0347	0.0164
SL vs. SI	88	59.37	30.62	0.602	1.485
SL vs. SB	38	36.87	26.87	0.0730	0.222
SN vs. SF1	118	54.37	45.62	0.00563	2.78
SN vs. SD	19	16.87	40.62	0.249	0.775
SN vs. SW	153	56.87	0.63	0.00113	0.0194
SN vs. SI	18	58.12	35.62	3.11	0.655
SN vs. SB	242	30.62	33.12	0.448	0.0788
SF1 vs. SD	24	24.37	100.60	0.00456	0.125
SF1 vs. SW	41	19.37	0.63	0.258	0.0294
SF1 vs. SI	88	63.12	119.40	0.0559	2.23
SF1 vs. SB	264	49.37	28.12	0.266	0.419
SD vs. SW	106	14.37	0.63	0.0587	0.0107
SD vs. SI	11	10.62	83.12	0.0854	0.625
SD vs. SB	100	40.62	10.62	0.156	0.239
SW vs. SI	5	211.90	0.625	0.309	0.0676
SW vs. SB	156	58.12	1.88	1.21	0.0400
SI vs. SB	375	40.62	59.37	0.8065	0.987

### 3.4 Genetic connectivity

At the smaller intra‐European scale, and based on microsatellite data, estimated population subdivision times were much smaller and in the order of decades to a few hundred years (Table 3B). The deepest inferred splits were between the Faroe Islands versus Brittany and southern Norway versus Brittany over 200 years in both cases. Inferred present‐day effective population sizes were again small and in the order of a few to a few dozen sporophytes with the exception of the Faroe Islands in its comparison with Denmark (101 sporophytes), and Ireland in its comparison with the Wadden Sea (212 sporophytes) (Table 3B). Inferred migration rates were low and typically well below one effective one‐way migrant per year, with a few exceptions including various connections among the samples from Denmark, Faroe Islands, Ireland, northern and southern Norway (2N_e_m ranging from 1.485 to 3.11), as well as from Brittany to the Wadden Sea (2*N*_e_ *m* = 1.214).

### 3.5 Comparing equilibrium and nonequilibrium inferences

**Figure 5:**
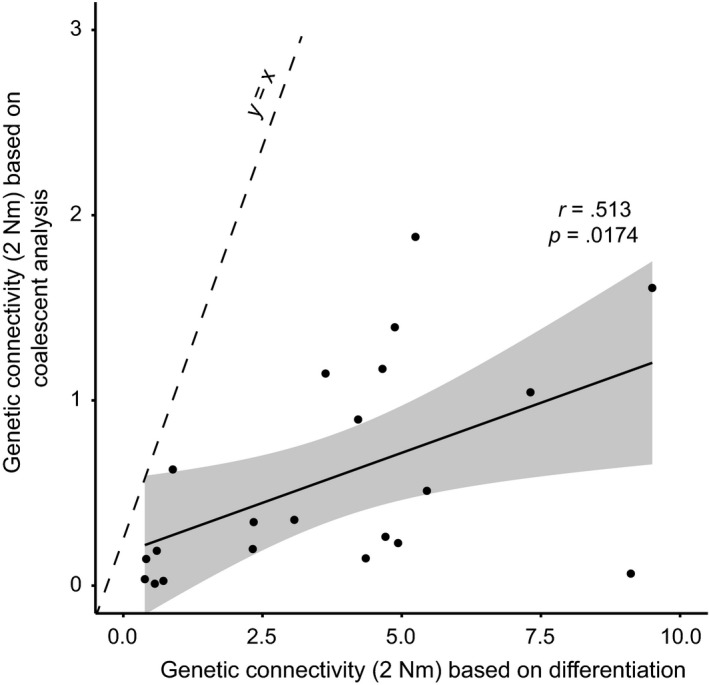
Genetic connectivity estimated using coalescent isolation‐with‐migration analyses versus traditional approach based on Wright's island model; the former does not and the latter does assume that equilibrium between isolation and migration has been reached. Dashed line indicates *x* = *y*; solid line is linear regression trend line with 95% confidence interval (gray area)

Genetic connectivity was estimated in two different ways: One assumes that equilibrium between isolation and migration has been reached since population subdivision (Wright's island model); the other does not make this assumption (coalescent analysis). A significant correlation was found (*r *=* *.5129; *p *=* *.017424) between connectivity estimates assuming equilibrium and those not assuming equilibrium (Figure 5). Genetic connectivity estimates not assuming equilibrium between isolation and migration were consistently lower.

## 4 DISCUSSION

*Saccharina latissima* populations at the European locations studied here are virtually unconnected by ongoing gene flow (Figure 4, Table 3B), which confirms results from earlier studies (Guzinski et al., 2016; Nielsen et al., 2016). Moreover, coalescent analyses show that effective dispersal is even lower than was concluded previously based on the assumption that equilibrium between isolation and migration has been reached (Figure 5). This implies that the observed levels of genetic differentiation are expected to increase even further in the future, unless exchange between natural kelp stands should increase. The latter could, for example, occur as a result of cultivation activities, as has happened in, for example, *S. japonica* in its native range in Asia (Zhang et al., 2015).

The pairwise migration estimates not assuming equilibrium differentiation are all well below the line of unity in Figure 5. If the studied populations of *S. latissima* had been in differentiation equilibrium, the data would have been expected to cluster around the line of unity. Given enough time, newly established populations will reach equilibrium levels of differentiation that are the result of a balance between isolation, mutation, lineage sorting, and gene flow. In 1984, it was shown by Crow and Aoki that the time for *F*_ST_ (a measure of population genetic differentiation) to reach half its equilibrium value theoretically equals ln2/(2*m* + 1/(2*N*_e_)), where *m* equals migration rate and *N*_e_ effective population size. In other words, the more migration, the faster is the approach to equilibrium, and the larger the population is, the slower that approach should be. Faurby and Barber (2012) demonstrated that at least theoretically, measured F_ST_ values depend at least as much on being in a nonequilibrium state as they do on gene flow. We have shown here for the kelp *S. latissima* that indeed, the observed level of genetic differentiation among populations is partly determined by being in this nonequilibrium state. The novel approach presented here may also be useful for other taxa in relatively recently (e.g., postglacially) colonized areas.

The conclusion that population differentiation of *S. latissima* has not yet reached its maximum in Europe is further emphasized by the fact that COI is not differentiated in northern Europe, while this is the case for the microsatellite DNA markers studied. This may seem counter‐intuitive, because mitochondrial haplotypes are theoretically expected to coalesce faster than nuclear alleles (Palumbi, Cipriano, & Hare, 2001). However, it can likely be explained by the fact that stochasticity among loci is expected to be significant. Furthermore, using multiple independent loci to infer past evolutionary processes should lead to better estimates than the single linked molecule of the mitochondrion (Hudson & Turelli, 2003).

Physical barriers in the form of long stretches of coastline with no hard‐bottom substrates and of deeper waters between the European mainland and islands are apparently not bridged by the potential dispersal capacities provided by floating sporophyte fragments and drifting meiospores. This is in accordance with studies on other macroalgae (Fredriksen et al., 1995; Gaylord et al., 2006) as well as other hard‐bottom taxa (Johansson, Banks, Glunt, Hassel‐Finnegan, & Buonaccorsi, 2008). Also, effective population sizes of *S. latissima* in the European part of its distribution are estimated by the coalescent approach to be small. This may mean that effective population sizes are indeed small, which seems reasonable for, for example, the location sampled in the western Wadden Sea consisting of a limited number of sporophytes which settled on a man‐made flood‐control dike. Alternatively, inferred small effective population sizes may result from past bottlenecks and/or recurring population size fluctuations, which is more likely to be the case for locations which at present house vast kelp forests such as the Norwegian sites and Brittany (Bekkby, Rinde, Erikstad, & Bakkestuen, 2009; Billot et al., 2003). Third, inbreeding is typically possible in kelp species and a factor in lowering the number of effectively interbreeding individuals (Coleman, 2013; Johansson et al., 2013). Inbreeding is likely to be a key factor in the western Wadden Sea location, because it houses such a small number of sporophytes. The high F_IS_ value observed for the microsatellite data for that site corroborates this (Table 1). Interestingly, inbreeding does not necessarily lower fitness‐related traits in kelp, probably because of purging of deleterious alleles during the free‐living haploid life stages (Barner, Pfister, & Wootton, 2011). Finally, high variance in reproductive success among individuals, typical of marine taxa with high fecundity, may also contribute to lower levels of genetic diversity within populations (and hence effective population size) than if reproductive success were more constant among individuals (Alberto et al., 2010; Hedgecock, 1994).

A hypothesis for the phylogeography of *S. latissima* was formulated by McDevit and Saunders (2010) on the basis of COI (cytochrome‐*c*‐oxidase 1, mitochondrial DNA) and ITS (Internal Transcribed Spacer, nuclear DNA) sequences. They suggest that an initial circumpolar distribution of the species was compressed to the south during early glaciation episodes leading to isolation. From this state of isolation, recolonization of the northeast Pacific and the northwest Atlantic would have occurred postglacially from a European source. Finally, contact is thought to have reestablished and to have resulted in hybridization in the Canadian Arctic and perhaps also the northwest Atlantic (see figs. 10‐12 in McDevit and Saunders, 2010). The minimum spanning network and coalescent analyses presented here provide support for parts of this hypothesis but not others. Firstly, *S. latissima*'s close relatives *S. japonica* and *S. cichorioides* (both originating from Japanese and other east Asian waters) are found to be genetically closest to those *S. latissima* that are at present found in the northeast Pacific (Figures 2 and 3). This makes it unlikely that the present‐day populations of *S. latissima* in the northeast Pacific are the result of colonization from Europe. However, it must be noted that the samples used for this inference were not taken based on a sampling regime specifically designed to infer species phylogeography. Large stretches of the distribution range around North America as well as Asia remain unstudied, and including these in future studies is expected to bring further insights into the history of the taxon. In addition, our estimates of the timing of population subdivision come with many uncertainties (particularly for microsatellites, see Section 2.1), but the estimates from COI coalescent analyses of more than a million years (Table 3A) makes postglacial transatlantic colonization highly unlikely, even if they were substantial overestimates. More consistent with the new analyses presented here is that transatlantic colonization of *S. latissima* happened before or during the Pleistocene and that isolated populations have survived glacial cycles on both sides of the north Atlantic. The direction of transatlantic colonization suggested by McDevit and Saunders (2010) to have been from Europe to North America is corroborated by the pattern seen in the minimum spanning network (Figure 3). Finally, Bolton (2010) argues that the origin of the genus *Saccharina* lies in the temperate northwest Pacific, because that is the center of species diversity of the genus, and that colonization of the Atlantic happened after the opening of the Bering Strait 5.5 Ma. Speciation within the genus in the Atlantic must have occurred after that although species status of several named species is still under debate and it has been argued that *Saccharina* is better seen as a large Pacific‐Atlantic species complex with ample morphological diversity (Bartsch et al., 2008 and references therein). The latter ties in to our observation that what is known as *S. cichorioides* with a temperate Asian distribution is so similar in its COI sequence that we conclude that it belongs to *S. latissima* (Figure 3). This was also suggested by Lane, Mayes, Druehl, and Saunders (2006) in their taxonomic revision of the order Laminariales after noting that its ITS sequence was identical to that of several other *Saccharina* species, but they nevertheless conservatively kept *S. cichorioides* as a separate species. We now hypothesize, because COI sequences of *S. cichorioides* and Pacific *S. latissima* are highly similar that *S. cichorioides* and *S. latissima* are best regarded as conspecific.

Summarizing, the new data and analyses presented here lead to the following updated hypothesis for the origin and historical distribution of *S. latissima*. It originated in the colder waters of the Asian Pacific and colonized the northeast Pacific by the end of the Miocene. After the opening of the Bering Strait, it colonized the north Atlantic across the Arctic, initially forming a continuous circumpolar range. During this time, some speciation has occurred, for example, *S. groenlandica* (McDevit & Saunders, 2010), while the status of some described species is still in need of molecular examination (Lane et al., 2006). The sequence of colonization was from the northeast Pacific to the European Atlantic and from there across the Atlantic Ocean to the Atlantic coasts of North America. The Pleistocene ice ages caused compression of isolation populations in southern regions on both sides of the Atlantic and along northeast Pacific coasts; all three of these isolated subregions have extant descendent populations today, with hybridization (or population mixing, depending on the taxonomic view of the species complex) in the postglacially recolonized Canadian Arctic.

A historical signature of recolonization of the European part of the distribution can also be inferred from our analyses, although the timing of events is less clear (Table 3B) because mutation rates of microsatellite markers are too variable and uncertain (see Section 2.1). We decided not to carry out detailed coalescent isolation‐with‐migration simulations using COI data at the intra‐European scale because of the shallowness of the gene tree. However, the comparison between the samples from Brittany and the Faroe Islands (Table 3A) provides an estimate of 174,000 years ago for the oldest population divergence among extant European *S. latissima*. This estimate is consistent with population expansion in Europe dating back to the ice ages of the Pleistocene and survival in northern refugia during the LGM (Last Glacial Maximum) and congruent with knowledge on postglacial recolonization of European Atlantic coasts (Maggs et al., 2008; Santos, Cruzeiro, Olsen, van der Veer, & Luttikhuizen, 2012). We suggest that haplotypes a, b, d, and e (Figure 3) are ancestral in Europe (being the closest relatives of the Pacific haplotypes) and have given rise to the outer Atlantic haplotypes g and r 100 to 200 thousand years ago. Crossing of the Atlantic Ocean was performed by ancestors of those outer Atlantic haplotypes. The present‐day inhabitants of Brittany and the western Wadden Sea form a separate group that derives from ancestral haplotypes. Summarizing, we hypothesize that during the ice ages *S. latissima* was compressed to the south in the northeast Atlantic but with glacial northern refugia. The western Wadden Sea houses a small population which is the result of a natural colonization event from the English Channel, but it is currently genetically isolated from it. Present‐day exchange among the locations studied is virtually nonexistent. Therefore, further research should be conducted regarding acceptable distances for transporting *S. latissima* from wild stands to cultivation sites without disturbing the natural genetic composition (Stévant, Rebours, & Chapman, 2017).

In conclusion, the populations of *S. latissima* as we know them today demonstrate a clear and consistent signature of a deep biogeographic history. If we are to maintain and make use of *S. latissima*'s strongly substructured natural genetic variation, aquaculture should not transport this species too far from its natural stands. The genetic variation contained within these populations is strongly spatially structured and is expected, if left undisturbed, to become even more differentiated in the future. The use of our novel approach of comparing connectivity derived from coalescent analysis with that derived from genetic differentiation (Figure 5) has proven an effective tool to determine whether equilibrium population differentiation has been reached or whether populations are expected to differentiate further in the future. This approach may be particularly relevant for species inhabiting recently colonized terrain and/or with large effective population sizes.

## CONFLICT OF INTEREST

None declared.

## AUTHOR CONTRIBUTIONS

PCL, FHMH, JDLB, and KT designed the study, FHMH and CR carried out the fieldwork, HJW performed the genotyping procedures, PCL did the bioinformatics analyses, and PCL wrote the manuscript with input from all coauthors. All authors gave final approval for publication.

## DATA ACCESSIBILITY

All data presented in this paper can be found on Genbank (accession numbers MF447855–MF447861) and in the additional files (microsatellite markers tested and microsatellite data).
