# Supplementary figures and images for: Strong population structure but no equilibrium yet: Genetic connectivity and phylogeography in the kelp Saccharina latissima (Laminariales, Phaeophyta)
Source: Ecol Evol. 2018 Apr 2;8(8):4265–77. doi: 10.1002/ece3.3968 (PMC5916297; doi:10.1002/ece3.3968)

Fig. S1

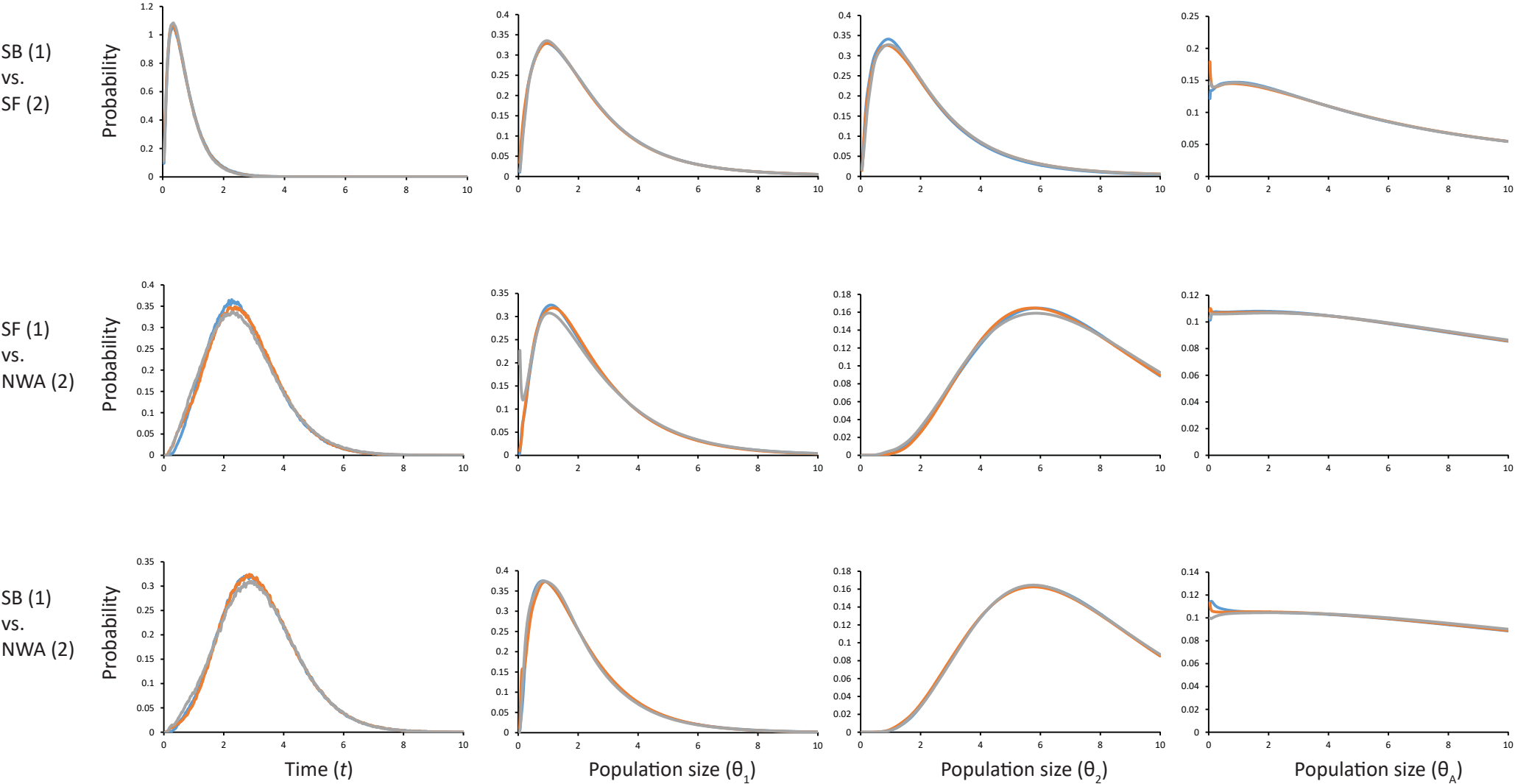

Supplement: Supplementary file 1 [file ECE3-8-4265-s001.pdf]
