# Supplementary material for: Strong population structure but no equilibrium yet: Genetic connectivity and phylogeography in the kelp Saccharina latissima (Laminariales, Phaeophyta)
Source: Ecol Evol. 2018 Apr 2;8(8):4265–77. doi: 10.1002/ece3.3968 (PMC5916297; doi:10.1002/ece3.3968)
Supplement: Supplementary file 2 [file ECE3-8-4265-s002.pdf]

marker	repeat	repeat_type	SRR305166_readno	primer_f	primer_r	Tm_f	Tm_r	expected_fragment_size	$N_{alleles}$	size range (bp)
Lat01	(AT)8	di-repeat	100157	TCGTCATTAGGGACACGCC	TGACGCGTTTCCATTTCGG	60.23	59.87	301		
Lat02	(AG)6	di-repeat	191744	AAGATCACGGAGGCGATCC	TCACTCACTGCGAAGGTGG	60.01	60.38	242		
Lat03	(AT)8	di-repeat	179145	ATAACTTGCCGCCACGGAG	GGTACTCGAATGCACACTCTTG	61.2	60.08	231		
Lat04	(AT)7	di-repeat	210618	TCCATCCTTCATGGCGGTC	ACGTTCTGCAGCTTTGACG	60.23	59.79	351		
**Lat05**	**(AT)6**	**di-repeat**	**213571**	**GGTTCGGCCTTTGCTAGTC**	**GTTGCTGCCTTCCTAAGCG**	**59.27**	**59.94**	**176**	**2**	**192-194**
Lat06	(AT)6	di-repeat	293080	GAATGGACAACGGCCACAG	AGGACGGAACCGACCATAC	59.86	59.56	397		
Lat07	(GCT)6	tri-repeat	160768	ACGGTTGGTTACAGTCGGG	CCGGAACATCACGTCAAGC	60.38	59.94	290		
**Lat08**	**(GGT)4**	**tri-repeat**	**236061**	**GTGGCTGTAGACCGGGTAG**	**AAGAAGCGCCGCAAAGTTC**	**59.93**	**60.45**	**375**	**3**	**389-394**
**Lat09**	**(AGC)8**	**tri-repeat**	**210681**	**CGCCCTTGTGAATCTCAGC**	**GCCATCTTCAAGTCACCAACG**	**59.64**	**60.55**	**220**	**6**	**229-244**
Lat10	(GAT)4	tri-repeat	130020	TCGTGTGAAGGGTTGAGGC	TGTCACTCACTGCGAACCC	60.68	60.68	225		
Lat11	(AAC)4	tri-repeat	329446	GCGCCAACGATCAGTATCC	TCAGTGGCCTGTCACTACG	59.50	59.78	171		
Lat12	(GGT)7	tri-repeat	307144	TACGGTAGAACGGTCGCTG	GTGTCACTCACTGCGAAGC	59.94	59.87	297		
Lat13	(CT)6	di-repeat	131242	CCGTCGCACGTATTTGGTC	CACTGCGAAGGGCAGATAAC	60.01	59.73	212		
Lat14	(CA)6	di-repeat	132283	CCCTACCACTTCGAGTGCC	GTACGTCCTTCGGGACACC	60.53	60.53	235		
**Lat15**	**(CA)6**	**di-repeat**	**135701**	**ACGGGTAGAAAGGACGGTG**	**TACGCTGTAGAGCAGTCCG**	**59.78**	**59.64**	**150**	**5**	**163-171**
Lat16	(GA)7	di-repeat	137304	CGGAGGATCCCAGATGGC	CTGTCACTCACTGCGAAGC	60.01	59.58	348		
Lat17	(TA)9	di-repeat	140102	CCCTTGGACCTTGGACAAC	GTACCGGGAACGGGAAACC	58.72	61.12	253		
**Lat18**	**(TA)5**	**di-repeat**	**143539**	**TTTCACATCTCGGACGGGC**	**TGCGCAGGATCTGTTCAGG**	**60.83**	**60.83**	**254**	**4**	**271-277**
**Lat19**	**(GT)13**	**di-repeat**	**147678**	**GAAACGTGCTAGACGCTGG**	**GTACGATTGCGTCCATGCC**	**59.65**	**60.09**	**169**	**27**	**174-233**
Lat20	(GC)6	di-repeat	152716	GGGCCAAGCATTGCGATAG	TTAAGGGTCCCGGAAAGCG	60.08	60.46	311		
Lat21	(CT)13	di-repeat	157991	AGCTTCTTTGGGCGGTTTC	CCTCGTGTGGCCTTCGG	59.41	60.82	312		
Lat22	(CG)6	di-repeat	280958	CCGTCCGGTTACCTTTGAC	TCTGTCGCCTTTCGACGC	58.91	61.15	282		
Lat23	(CAG)5	tri-repeat	110848	GCTGTGAGCTCGAGGATTTC	TACGGAGTACGTTCGGTGG	59.45	59.57	387		
Lat24	(CGT)6	tri-repeat	142768	GACCCTCCCGTACGAAACC	CCTACCACCAGTACGGAACG	60.53	60.57	340		
**Lat25**	**(TGC)6**	**tri-repeat**	**160375**	**CGCGTGTTACCATCGTCAG**	**TCTTCGCTGTTCCGAGGAC**	**59.73**	**60.16**	**192**	**2**	**219-223**
Lat26	(TGC)6	tri-repeat	172368	CTGGTCGTCTGCATTCTGG	CAGTTTCGTCGCCTACTGC	58.98	59.65	259		
Lat27	(GAT)4	tri-repeat	180792	AGCTGTCTTGACCTGGCG	GACGAAAGCAGGAAAGGTCG	60.40	59.94	242		
Lat28	(GAC)3	tri-repeat	192623	CGGATACGCTACCGACGAG	TGCGAATGGCGGTAGATCG	60.52	61.04	321		
Lat29	(TGC)7	tri-repeat	212432	CTGCTGCTCGCCATTCATC	GTCACTCACTGCGAAAGGG	60.09	59.20	151		
Lat30	(GCT)4	tri-repeat	224821	GGGCAGTGGTCGTAGATCC	CACCATTCGGCTGCAAGTC	60.31	60.23	281		
Lat31	(CAG)3	tri-repeat	253424	CGTACGTGCCAACGATGAG	TCCACTCACTGCGAAGAGC	59.73	60.45	389		
Lat32	(GCT)6	tri-repeat	301446	CGGAAGATCCACGCGATTG	CACACGAACAAGTACCGGC	59.80	59.87	316		

**Lat33**	**(CT)6**	**di-repeat**	**201581**	**ATCCCGTCTTTCTCGGTGG**	**GTGGCTCGCGATTTCAAGG**	**59.86**	**60.30**	**176**	**3**	**195-199**
**Lat34**	**(AT)6**	**di-repeat**	**203232**	**AGAACAAGACCGCTCAGGC**	**AGGGCGGTTCTTCTACGAG**	**60.75**	**59.56**	**229**	**7**	**238-250**
Lat35	(TA)10	di-repeat	283134	TGTCAAGGACCGCTTTGGG	CTCACTGCGAAGAGCCAAC	60.99	59.57	160		
Lat36	(GA)10	di-repeat	231456	GGTTCGAATCCCAGCTACG	CCTCGGTCAGTCTCAGTGG	58.76	59.86	292		
**Lat37**	**(GT)7**	**di-repeat**	**278229**	**GTGGTGCGCAGGAAAGTG**	**GACCACCTTGAGACCTCCG**	**60.09**	**60.16**	**330**	**6**	**348-358**
Lat38	(TGC)5	tri-repeat	208821	AGCCAGTAACCGACGAAGG	GTCCCGGTTCGATAAACGC	60.16	59.72	275		
Lat39	(GCT)8	tri-repeat	208854	TCTTCTCGACCCACTCGTTG	CCTGTCACTCACTGCGAAC	60.15	59.21	191		
Lat40	(CGA)6	tri-repeat	240939	CACGCGACCTTTCTAAGCG	CTCACTGCGAAGTTGGTGC	60.02	60.16	166		
Lat41	(CAG)8	tri-repeat	241080	TGAGCCCGATAGCACCAAG	CCTCCGGAATAGCCACCTC	60.23	60.01	197		
Lat42	(GAT)6	tri-repeat	241630	ACTGGGTAAGGGAAGGATGC	TCTACGGAGGGTTATCGAGC	59.86	59.15	151		
