# Supplementary material for: Strong population structure but no equilibrium yet: Genetic connectivity and phylogeography in the kelp Saccharina latissima (Laminariales, Phaeophyta)
Source: Ecol Evol. 2018 Apr 2;8(8):4265–77. doi: 10.1002/ece3.3968 (PMC5916297; doi:10.1002/ece3.3968)
Supplement: Supplementary file 3 [file ECE3-8-4265-s003.pdf]

ind	lat05		lat08		lat09		lat15		lat18		lat19		lat25		lat33		lat34		lat37	
SB01	192	192	389	394	235	235	163	165	273	273	176	180	220	220	197	197	240	240	352	352
SB02	192	192	389	394	235	235	163	163	277	277	174	180	220	220	195	197	240	240	#N/A	#N/A
SB03	192	192	389	394	235	235	165	167	273	275	176	176	219	220	195	195	242	242	350	352
SB04	192	192	394	394	235	235	165	165	273	273	202	233	220	220	197	197	240	240	350	352
SB05	192	192	394	394	235	235	163	165	273	273	178	218	220	220	197	197	242	242	352	352
SB06	192	192	394	394	235	235	163	165	275	275	178	178	219	220	195	197	240	242	352	352
SB07	192	192	389	394	235	235	165	165	271	273	178	220	220	220	197	197	240	242	350	352
SB08	192	192	394	394	235	235	165	165	271	271	176	204	219	220	195	197	240	242	352	352
SB09	192	192	394	394	235	235	165	165	275	275	206	223	219	220	197	197	240	240	350	352
SB10	192	192	394	394	235	235	165	167	271	271	202	212	219	220	197	197	240	242	352	352
SB11	192	192	389	394	235	235	163	165	273	273	176	233	220	220	195	197	240	240	352	352
SB12	192	192	394	394	235	235	163	165	#N/A	#N/A	176	223	220	220	197	197	#N/A	#N/A	352	352
SB13	192	192	394	394	235	235	165	165	273	273	176	198	219	220	197	197	240	240	352	352
SB14	192	192	389	394	235	235	163	163	273	273	176	210	219	220	197	197	240	240	#N/A	#N/A
SB15	192	192	394	394	235	235	163	165	273	273	174	233	219	220	195	197	240	242	352	352
SB16	192	192	394	394	235	235	165	165	273	273	176	223	220	220	197	197	240	242	350	352
SB17	192	192	394	394	235	235	165	165	275	277	176	176	220	220	195	197	240	240	352	352
SB18	192	192	394	394	235	235	163	165	271	271	178	212	220	220	197	197	240	240	352	352
SB19	192	192	394	394	235	235	165	165	273	273	178	178	220	220	197	197	240	242	352	352
SB20	192	192	394	394	238	238	165	165	273	273	185	187	220	220	197	197	238	244	352	358
SB20	192	192	394	394	235	235	#N/A	#N/A	273	273	180	218	220	220	197	197	240	242	352	352
SB21	192	192	389	394	235	235	163	165	#N/A	#N/A	176	176	219	220	195	197	242	242	352	352
SB22	192	192	389	394	235	235	163	165	273	273	176	220	219	220	195	197	240	240	350	352
SB23	192	192	394	394	235	235	163	165	273	273	176	178	220	220	197	197	240	240	352	352
SB24	192	192	394	394	235	235	165	165	271	277	178	225	220	220	195	197	240	242	350	352
SB25	192	192	389	394	235	235	167	167	273	273	176	178	219	220	195	197	240	240	352	352
SB26	192	192	389	394	235	235	165	165	273	273	206	208	220	220	197	197	240	240	350	350
SB27	192	192	394	394	235	235	163	165	#N/A	#N/A	176	178	220	220	197	197	240	242	#N/A	#N/A
SB28	192	192	394	394	235	235	163	165	271	271	180	210	219	220	195	197	242	242	352	352
SB29	192	192	394	394	235	235	165	165	273	273	178	178	220	220	195	197	240	244	352	352
SB30	192	192	394	394	235	235	165	165	#N/A	#N/A	178	180	220	220	197	197	240	240	352	352
SB31	192	192	394	394	235	235	165	165	273	273	178	223	220	220	197	197	240	242	352	352

ind	lat05		lat08		lat09		lat15		lat18		lat19		lat25		lat33		lat34		lat37	
SB32	192	192	389	394	235	235	165	167	273	273	174	178	220	220	195	197	240	240	352	352
SD01	192	194	394	394	235	235	165	165	273	273	180	193	220	220	195	195	242	244	352	352
SD02	194	194	394	394	235	238	165	167	273	273	195	204	220	220	#N/A	#N/A	#N/A	#N/A	352	352
SD03	192	192	394	394	235	235	165	167	273	273	180	189	220	220	#N/A	#N/A	#N/A	#N/A	#N/A	#N/A
SD04	192	194	394	394	235	235	165	165	#N/A	#N/A	183	198	220	220	#N/A	#N/A	#N/A	#N/A	#N/A	#N/A
SD05	192	194	394	394	235	235	163	165	273	273	180	193	220	220	197	197	244	244	352	352
SD06	194	194	394	394	235	235	165	165	273	273	191	195	220	220	197	197	244	244	352	352
SD07	192	194	394	394	238	238	165	165	273	273	180	180	220	220	195	197	#N/A	#N/A	#N/A	#N/A
SD08	192	192	394	394	235	238	163	165	273	273	193	195	220	220	197	197	#N/A	#N/A	352	352
SD09	192	192	394	394	235	235	163	165	273	273	178	180	220	220	195	197	244	244	352	352
SD10	194	194	394	394	238	238	163	165	273	273	180	195	220	220	#N/A	#N/A	244	244	352	352
SD11	#N/A	#N/A	394	394	235	238	163	165	#N/A	#N/A	180	202	220	220	#N/A	#N/A	#N/A	#N/A	352	352
SD12	192	194	394	394	235	235	167	167	273	273	#N/A	#N/A	220	220	197	197	#N/A	#N/A	#N/A	#N/A
SD13	194	194	394	394	235	238	163	163	273	273	180	193	220	220	197	197	#N/A	#N/A	#N/A	#N/A
SD14	194	194	394	394	235	238	165	165	273	273	189	191	220	220	#N/A	#N/A	#N/A	#N/A	#N/A	#N/A
SD15	192	194	394	394	235	235	165	165	273	273	176	180	220	220	197	197	#N/A	#N/A	352	352
SD16	192	192	394	394	235	238	165	165	273	273	178	198	220	220	197	197	#N/A	#N/A	352	352
SD17	194	194	394	394	235	235	165	165	273	273	180	193	220	220	195	195	244	244	352	352
SD18	194	194	394	394	235	238	165	165	273	273	189	198	220	220	#N/A	#N/A	244	244	352	352
SD19	192	194	394	394	235	235	165	165	#N/A	#N/A	180	180	220	220	#N/A	#N/A	244	244	#N/A	#N/A
SD20	192	194	394	394	238	238	163	163	273	273	176	180	220	220	195	197	244	244	352	352
SD21	194	194	394	394	235	238	163	163	273	273	180	193	220	220	197	197	244	244	352	352
SD22	192	192	394	394	235	235	165	165	#N/A	#N/A	198	198	220	220	#N/A	#N/A	#N/A	#N/A	352	352
SF01	192	194	389	394	235	235	165	165	273	273	180	204	220	220	197	197	244	244	352	352
SF02	194	194	394	394	229	235	165	165	273	275	185	185	220	220	197	197	244	244	352	352
SF03	192	194	394	394	232	235	165	165	273	273	183	185	220	220	197	197	244	244	352	352
SF04	194	194	394	394	235	235	163	163	273	273	183	204	220	220	197	197	244	244	350	352
SF05	192	192	389	394	235	241	165	165	273	273	180	183	220	220	197	197	242	244	352	352
SF06	192	194	#N/A	#N/A	235	238	165	165	273	273	178	185	220	220	197	197	244	244	352	352
SF07	192	194	389	394	235	238	165	165	#N/A	#N/A	178	183	220	220	197	197	244	244	352	352
SF08	192	194	394	394	232	235	165	165	273	273	183	189	220	220	197	197	244	244	352	352
SF09	192	194	394	394	235	238	165	165	273	273	180	183	220	220	197	197	244	244	352	354

ind	lat05		lat08		lat09		lat15		lat18		lat19		lat25		lat33		lat34		lat37	
SF10	192	194	389	394	235	238	165	165	273	273	183	183	220	220	197	197	244	244	350	352
SF11	192	194	394	394	235	244	163	165	273	273	180	185	220	220	197	197	244	244	350	352
SF12	194	194	394	394	235	238	165	165	273	273	183	183	220	220	197	197	244	244	352	352
SF13	192	192	394	394	235	238	163	165	273	273	183	185	220	220	197	197	244	244	352	352
SF14	192	192	394	394	235	235	165	165	273	273	180	183	220	220	197	197	244	244	352	352
SF15	192	194	394	394	232	235	165	165	273	273	183	185	220	220	197	197	244	244	352	352
SF16	192	194	394	394	235	238	163	165	273	273	178	178	220	220	197	197	242	244	352	352
SF17	192	194	394	394	235	238	165	165	273	273	183	187	220	220	197	197	244	244	352	352
SF18	#N/A	#N/A	394	394	235	235	165	165	273	273	180	183	219	219	197	197	244	244	352	352
SF19	#N/A	#N/A	394	394	235	235	163	165	273	273	178	187	219	219	197	197	244	244	352	352
SF20	#N/A	#N/A	394	394	235	235	165	165	273	273	185	185	219	219	197	197	238	244	352	352
SF21	#N/A	#N/A	394	394	235	235	165	165	273	273	180	185	219	219	197	197	244	244	348	352
SF22	#N/A	#N/A	394	394	235	235	165	165	273	273	180	185	219	219	197	197	242	244	348	352
SF23	#N/A	#N/A	394	394	235	235	163	163	273	273	180	204	219	219	197	197	244	244	352	352
SF24	#N/A	#N/A	394	394	235	235	165	165	273	273	185	200	219	219	197	197	244	244	350	352
SF25	#N/A	#N/A	394	394	235	235	165	165	273	273	185	195	219	219	197	199	238	244	348	354
SF26	#N/A	#N/A	394	394	235	235	165	165	273	273	180	180	219	219	197	197	244	244	352	358
SF27	#N/A	#N/A	394	394	235	235	165	165	273	273	180	180	219	219	197	197	244	244	350	352
SF28	#N/A	#N/A	394	394	235	235	165	165	273	273	180	187	219	219	197	197	244	244	350	352
SF29	#N/A	#N/A	394	394	235	235	165	165	273	273	178	185	219	219	197	197	244	244	352	354
SF30	#N/A	#N/A	394	394	235	235	163	163	273	273	180	185	219	219	197	197	244	244	352	352
SF31	#N/A	#N/A	394	394	235	235	163	165	273	273	178	193	219	219	197	197	242	244	350	352
SF32	#N/A	#N/A	394	394	235	235	163	165	273	273	185	187	219	219	195	199	244	244	354	354
SI01	192	192	394	394	235	235	163	165	273	273	180	193	220	220	197	199	242	242	#N/A	#N/A
SI02	192	192	392	394	238	238	165	167	273	273	178	187	220	220	197	197	242	244	352	352
SI03	192	192	394	394	235	238	163	165	273	273	178	180	220	220	197	197	242	242	352	352
SI04	192	192	394	394	235	235	165	165	273	273	180	183	220	220	197	197	242	242	350	352
SI05	192	192	389	394	238	238	165	165	273	273	183	185	219	220	197	197	242	242	352	352
SI06	192	192	394	394	238	238	165	165	273	273	183	191	220	220	197	197	242	244	352	352
SI07	192	192	394	394	235	235	163	163	273	273	187	198	220	220	197	197	244	244	352	352
SI08	192	192	394	394	238	238	165	171	273	273	180	185	220	220	197	197	242	244	352	352
SI09	192	192	394	394	238	238	163	163	273	273	178	183	220	220	197	197	242	244	350	352

ind	lat05		lat08		lat09		lat15		lat18		lat19		lat25		lat33		lat34		lat37	
SI10	192	192	394	394	238	238	163	165	273	273	183	185	220	220	197	197	242	244	350	352
SI11	192	192	394	394	235	238	163	165	273	273	178	187	220	220	197	197	242	244	352	352
SI12	192	192	394	394	235	238	163	165	273	273	180	185	220	220	197	197	238	244	352	352
SI13	192	192	394	394	235	235	163	165	273	273	185	187	220	220	197	197	242	242	350	352
SI14	192	192	394	394	235	238	163	165	273	273	180	198	220	220	197	197	238	242	350	350
SI15	192	192	394	394	235	238	163	165	273	273	183	183	219	220	197	197	242	242	352	352
SI16	192	192	394	394	235	238	167	169	273	273	178	187	220	220	197	197	242	244	352	352
SI17	192	194	389	394	235	238	165	165	273	273	189	198	220	220	197	197	242	242	352	352
SI18	192	192	394	394	235	238	165	165	273	275	180	189	220	220	197	197	242	242	350	352
SI19	192	192	389	394	235	238	165	165	273	273	183	187	220	223	197	197	242	242	352	352
SI20	192	192	394	394	235	238	165	165	273	273	191	202	220	220	197	197	244	250	352	352
SI21	192	192	394	394	235	235	165	165	273	273	178	185	220	220	197	197	242	250	352	352
SI22	192	192	394	394	235	235	163	165	273	273	180	180	220	220	197	197	242	244	352	352
SI23	192	192	394	394	235	238	163	165	273	273	180	185	220	220	197	197	238	244	352	352
SI24	192	192	394	394	235	238	165	167	273	273	183	185	220	220	195	197	242	242	352	352
SI25	192	192	394	394	235	238	165	165	273	273	185	187	220	220	195	197	242	242	350	352
SI26	192	192	394	394	235	238	165	165	273	275	185	189	220	220	197	197	242	246	352	352
SI27	192	194	394	394	238	238	163	165	273	273	187	189	220	220	197	197	242	242	352	352
SI28	192	192	394	394	235	238	165	165	273	273	178	183	220	220	197	197	242	242	352	352
SI29	192	192	389	389	238	238	163	165	273	273	185	185	220	220	197	197	242	244	352	352
SI30	192	192	394	394	238	238	163	165	273	273	183	187	220	220	195	197	242	242	352	352
SL01	192	192	394	394	235	235	163	165	273	273	180	185	219	220	197	197	244	244	352	352
SL02	192	192	394	394	235	235	163	165	273	273	183	185	219	220	195	197	242	244	352	358
SL03	192	192	394	394	235	238	163	165	273	273	180	187	219	220	197	199	244	244	352	352
SL04	192	192	394	394	235	244	165	165	273	273	180	183	219	220	197	197	244	244	352	352
SL05	192	192	394	394	235	238	165	165	273	273	178	180	220	220	197	197	242	244	352	358
SL06	192	192	394	394	235	238	#N/A	#N/A	#N/A	#N/A	185	206	219	220	197	197	238	244	352	358
SL07	192	192	394	394	232	235	165	165	273	273	185	200	219	220	197	197	244	244	352	352
SL08	192	192	394	394	235	238	#N/A	#N/A	273	273	185	187	219	219	#N/A	#N/A	244	244	#N/A	#N/A
SL09	192	192	394	394	235	238	165	165	273	273	180	187	220	220	195	197	242	244	352	352
SL10	192	192	394	394	235	235	165	165	273	273	185	206	220	220	195	197	244	244	352	358
SL11	192	192	394	394	235	235	163	165	273	273	176	189	220	220	197	197	244	244	352	352

ind	lat05		lat08		lat09		lat15		lat18		lat19		lat25		lat33		lat34		lat37	
SL12	192	192	394	394	235	238	165	165	273	273	180	183	219	220	195	197	242	244	352	352
SL13	192	194	394	394	235	235	165	165	273	273	180	206	220	220	197	197	242	244	352	358
SL14	192	192	394	394	235	238	163	165	273	273	180	183	220	220	197	199	242	244	352	354
SL15	192	192	394	394	235	238	163	163	273	273	178	183	219	220	195	197	244	244	352	352
SL16	192	194	394	394	238	238	165	165	273	273	185	208	219	220	197	197	244	244	352	352
SL17	192	192	394	394	238	238	#N/A	#N/A	#N/A	#N/A	178	180	220	220	197	197	#N/A	#N/A	352	352
SL18	192	192	394	394	235	238	165	165	#N/A	#N/A	180	206	220	220	195	197	238	242	352	358
SL19	192	192	394	394	235	238	165	165	273	273	180	202	219	219	197	197	242	244	352	352
SL20	192	192	394	394	235	238	165	165	273	273	180	208	219	220	197	197	244	244	#N/A	#N/A
SL21	192	194	394	394	235	235	165	165	273	273	180	185	219	220	197	197	242	244	352	352
SL22	192	192	394	394	235	235	165	165	273	273	180	183	219	220	197	199	244	244	352	352
SL23	192	192	394	394	235	238	165	165	273	273	180	185	219	220	197	197	238	242	352	358
SL24	192	192	394	394	235	238	163	165	273	273	180	187	219	219	197	199	242	244	352	352
SL25	192	192	394	394	235	235	165	165	#N/A	#N/A	180	180	220	220	197	197	238	242	358	358
SL26	192	192	394	394	235	235	165	165	273	273	178	183	220	220	195	197	242	242	350	352
SL27	192	192	394	394	235	235	163	165	273	273	180	185	219	220	195	197	242	246	350	352
SL28	192	192	394	394	235	235	165	165	273	273	185	187	219	220	197	197	242	244	352	358
SL29	192	192	394	394	238	238	165	165	273	273	180	180	220	220	197	199	242	244	352	358
SL30	192	192	394	394	235	235	165	165	#N/A	#N/A	178	180	219	220	195	199	244	250	358	358
SL31	192	192	394	394	238	244	165	165	273	275	174	180	219	219	197	199	244	244	352	358
SN01	192	192	394	394	235	235	165	165	273	273	180	202	220	220	195	197	238	244	352	352
SN02	192	192	394	394	235	235	165	165	#N/A	#N/A	180	202	220	220	195	197	238	244	#N/A	#N/A
SN03	192	192	394	394	235	235	163	165	273	273	185	193	220	220	195	197	244	248	352	352
SN04	192	192	394	394	235	235	163	167	273	273	180	200	220	220	195	197	244	244	350	352
SN05	192	192	394	394	235	235	165	165	273	273	180	185	220	220	197	197	244	244	352	352
SN06	#N/A	#N/A	394	394	#N/A	#N/A	165	165	273	273	183	206	220	220	195	197	244	244	352	352
SN07	192	192	394	394	235	235	163	165	273	273	180	187	220	220	195	197	244	244	352	352
SN08	192	192	394	394	235	238	163	165	271	273	180	183	220	220	195	197	238	242	352	352
SN09	192	192	#N/A	#N/A	235	238	165	165	273	275	195	198	220	220	197	197	242	244	352	358
SN10	192	192	394	394	235	238	165	165	275	275	183	185	220	220	195	197	244	244	352	352
SN11	192	192	394	394	235	238	163	163	273	273	183	187	220	220	197	197	244	244	352	352
SN12	192	192	394	394	235	238	165	165	#N/A	#N/A	185	187	220	220	195	197	244	244	352	352

ind	lat05		lat08		lat09		lat15		lat18		lat19		lat25		lat33		lat34		lat37	
SN13	192	192	394	394	235	238	163	163	273	273	183	185	220	220	197	197	244	244	352	352
SN14	192	192	394	394	235	235	165	165	273	273	187	187	220	220	197	197	238	244	#N/A	#N/A
SN15	192	192	394	394	235	235	163	167	273	273	183	183	220	220	197	197	242	250	352	358
SN16	192	192	394	394	235	235	163	165	271	271	185	198	220	220	195	195	244	250	352	358
SN17	192	192	394	394	235	238	165	165	273	275	187	187	220	220	197	197	238	250	352	352
SN18	192	192	394	394	238	238	163	165	273	273	180	180	220	220	197	197	244	244	352	352
SN19	192	192	394	394	235	235	165	165	271	275	183	185	220	220	195	197	238	238	352	352
SN21	192	192	394	394	235	235	165	165	273	275	185	185	220	220	197	197	244	244	352	352
SN22	192	192	394	394	235	235	165	165	#N/A	#N/A	181	187	220	220	195	195	238	238	352	352
SN23	192	192	394	394	235	235	165	165	273	273	180	187	220	220	195	195	242	244	352	352
SN24	192	194	394	394	235	238	165	165	#N/A	#N/A	180	180	219	220	195	197	244	244	352	352
SN25	192	192	394	394	235	235	165	165	273	273	178	185	220	220	195	197	244	244	352	352
SN26	192	192	394	394	235	235	#N/A	#N/A	273	273	178	183	220	220	195	197	244	244	#N/A	#N/A
SN27	192	192	394	394	235	235	165	165	273	273	180	185	220	220	197	197	238	244	352	352
SN28	192	192	394	394	235	235	163	165	273	275	183	185	220	220	197	197	242	244	352	352
SN29	#N/A	#N/A	394	394	235	235	165	165	275	275	180	185	220	220	195	195	244	244	352	352
SN30	192	192	394	394	#N/A	#N/A	163	165	#N/A	#N/A	185	189	#N/A	#N/A	195	195	244	244	352	352
SS01	#N/A	#N/A	394	394	235	238	165	165	273	273	180	185	219	219	197	197	238	244	352	352
SS02	#N/A	#N/A	#N/A	#N/A	235	235	165	165	273	273	178	180	219	219	197	197	242	244	352	352
SS03	#N/A	#N/A	394	394	235	238	165	165	273	275	178	187	219	219	197	197	242	244	352	352
SS04	#N/A	#N/A	389	394	235	238	165	165	273	273	176	191	219	219	197	197	242	242	352	352
SS05	#N/A	#N/A	394	394	235	238	165	165	273	275	180	185	219	219	197	199	242	244	352	352
SS06	#N/A	#N/A	394	394	235	235	165	165	273	273	178	180	219	219	197	197	242	244	352	352
SW01	192	192	389	394	235	235	165	165	#N/A	#N/A	176	176	219	219	197	197	242	242	352	352
SW02	192	192	389	394	235	235	165	165	275	275	176	176	219	219	197	197	242	242	352	352
SW03	192	192	389	389	235	235	165	165	#N/A	#N/A	176	176	219	219	197	197	242	242	352	352
SW04	192	192	389	394	235	235	165	165	#N/A	#N/A	176	176	219	219	197	197	242	242	352	352
SW05	192	192	389	389	235	235	165	165	#N/A	#N/A	176	176	219	219	197	197	242	242	352	352
SW06	192	192	389	389	235	235	165	165	#N/A	#N/A	176	176	219	219	197	197	242	242	352	352
SW07	192	192	389	394	235	235	165	165	#N/A	#N/A	176	176	219	219	197	197	242	242	352	352
SW08	192	192	389	394	235	235	165	165	275	275	176	176	219	219	197	197	242	242	#N/A	#N/A
SW09	192	192	389	389	235	235	165	165	275	275	176	176	219	219	197	197	242	242	352	352

ind	lat05		lat08		lat09		lat15		lat18		lat19		lat25		lat33		lat34		lat37	
SW10	192	192	#N/A	#N/A	#N/A	#N/A	#N/A	#N/A	#N/A	#N/A	176	176	#N/A	#N/A	197	197	242	242	#N/A	#N/A
SW11	192	192	389	389	235	235	165	165	275	275	176	176	219	219	197	197	242	242	352	352
SW12	#N/A	#N/A	389	389	235	235	165	165	275	275	176	176	219	219	197	197	242	242	352	352
SW13	#N/A	#N/A	#N/A	#N/A	235	235	#N/A	#N/A	#N/A	#N/A	176	176	219	219	197	197	242	242	352	352
SW14	192	192	#N/A	#N/A	235	235	163	165	#N/A	#N/A	176	176	219	219	#N/A	#N/A	#N/A	#N/A	#N/A	#N/A
SW15	192	192	#N/A	#N/A	235	235	#N/A	#N/A	#N/A	#N/A	176	176	219	219	197	197	242	242	#N/A	#N/A
SW16	192	192	389	389	235	235	165	165	#N/A	#N/A	176	176	219	219	197	197	242	242	352	352
SW17	#N/A	#N/A	#N/A	#N/A	235	238	165	165	#N/A	#N/A	176	176	219	219	197	197	242	242	#N/A	#N/A
SW18	192	192	389	394	235	235	#N/A	#N/A	#N/A	#N/A	176	176	219	219	197	197	242	242	352	352
SW19	192	192	394	394	235	235	165	165	275	275	176	176	219	219	197	197	242	242	352	352
SW20	192	192	394	394	235	235	165	165	275	275	176	176	219	219	197	197	242	242	#N/A	#N/A
SW21	192	192	389	394	235	235	165	165	275	275	176	176	219	219	197	197	242	242	352	352
SW22	192	192	389	389	235	235	165	165	275	275	176	176	219	219	197	197	242	242	352	352
SW23	192	192	394	394	235	235	165	165	275	275	176	176	219	219	197	197	242	242	352	352
SW24	192	192	389	389	235	235	165	165	275	275	176	176	219	219	197	197	242	242	352	352
SW25	192	192	394	394	235	235	165	165	275	275	176	176	219	219	197	197	242	242	352	352
SW26	192	192	394	394	235	235	165	165	275	275	176	176	219	219	197	197	242	242	352	352
SW27	192	192	394	394	235	235	165	165	275	275	176	176	219	219	197	197	242	242	352	352
SW28	192	192	394	394	235	235	165	165	275	275	176	176	219	219	197	197	242	242	352	352
SW29	192	192	394	394	235	235	165	165	275	275	176	176	219	219	197	197	242	242	352	352
SW30	192	192	394	394	235	235	165	165	#N/A	#N/A	176	176	219	219	197	197	242	242	352	352
