# Supplementary material for: Strong population structure but no equilibrium yet: Genetic connectivity and phylogeography in the kelp Saccharina latissima (Laminariales, Phaeophyta)
Source: Ecol Evol. 2018 Apr 2;8(8):4265–77. doi: 10.1002/ece3.3968 (PMC5916297; doi:10.1002/ece3.3968)
Supplement: Supplementary file 4 [file ECE3-8-4265-s004.pdf]

Table S3 - Haplotype frequencies for partial cytochrome-c-oxidase I sequences for *Saccharina* spp. at various sampling locations. For abbreviations of sampling locations see Fig. 2 and Table 1.

	SL	SN	SF	SS	SD	SW	SI	SB	ARC[*]	NEP[*]	NWA[*]	FA[*]	IR[*]	*S. c.*[**]	*S. j.*[**]	total
a					3											3
b					1											1
c						25										25
d							1									1
e	19	29	1	2	18		29	1				1	1			101
f								32								32
g			29	1												30
h									7		42					49
i											1					1
j											1					1
k									16	5						21
l											1					1
m									1							1
n									4							4
o										1						1
p											1					1
q											1					1
r												3				3
s														8		8
t															1	1
u															2	2
v															14	14
total	19	29	30	3	22	25	30	33	28	6	47	4	1	8	17	302

[*]) re-analized from McDevit & Saunders (2010); [**]) re-analyzed from Balakirev *et al.* (2012); *S. c.* = *Saccharina cichoriodes*; *S. j.* = *Saccharina japonica*.
