# Supplementary material for: Strong population structure but no equilibrium yet: Genetic connectivity and phylogeography in the kelp Saccharina latissima (Laminariales, Phaeophyta)
Source: Ecol Evol. 2018 Apr 2;8(8):4265–77. doi: 10.1002/ece3.3968 (PMC5916297; doi:10.1002/ece3.3968)
Supplement: Supplementary file 5 [file ECE3-8-4265-s005.docx]

**Additional files**

Additional file 1: Table S1. Details for 42 microsatellite markers developed and tested from Genbank transcriptome archive SRR305166 for *Saccharina latissima*. The ten loci used for genotyping are in bold; primer_f = forward primer sequence; primer_r = reverse primer sequence; Tm_f, Tm_r = melting temperature of forward and reverse primer, respectively; N_alleles_ = number of alleles observed. (PDF 62 kb)

Additional file 2: Table S2. Microsatellite genotyping data for 213 *Saccharina latissima* individuals. (PDF 33 kb)

Additional file 3: Table S3. Haplotype frequencies for partial cytochrome-c-oxidase I sequences for *Saccharina* spp. at various sampling locations. For abbreviations of sampling locations see Fig. 2 and Table 1.

Additional file 4: Figure S1 - Marginal posterior probability distributions for coalescent analyses of population divergence. Three pairwise comparisons were made: SB (Brittany) versus SF (Faroe Islands), SF versus NWA (northwest Atlantic), and SB versus NWA. Migration rates were set to zero. Line colors represent three independent simulation runs; A = simulated ancestral population.
